# Irregular Menstruation and Treatment

**Published:** 1904-07

**Authors:** 


### IRREGULAR MENSTRUATION AND TREATMENT.

Practitioners of medicine are consulted by no class of patients who display greater solicitude than those who have amenorrhea.

In the popular mind failure of the menses to appear is supposed to be due either to pregnancy or tuberculosis, and either may cause a degree of anxiety that is truly intense.

The term amenorrhea is used to mean the total absence of the menstrual discharge, or a marked deficiency in the quantity of the flow. Amenorrhea may be physiological or pathological. During pregnancy the absence of the menstrual discharge is, of course, physiological and demands no consideration in this article. When pathological, the causes of amenorrhea may be said in general to be due to the following:

(1) Taking cold, at or near the menstrual epoch. (2) Severe mental perturbation, as fright, sorrow, or great elation of spirit. (3) It may be symptomatic in several affections, as tuberculosis, anmia, chlorosis, syphilis, typhoid fever, nephritis, pelvic peritonitis, and other morbid conditions. (4) Obesity. (5) Luxurious life, or overtaxing the nervous system. (6) Stenosis or atresia of the cervical canal, or imperfect development of the tubes, ovaries or uterus. (7) Vicarious menstruation may make the condition obscure, there being a discharge at the regular monthly periods from the nose, lungs, bladder, stomach, nipple, or other part.

The treatment of amenorrhea must comprehend attention to general considerations, and special indications must be remembered in the various expressions of amenorrhea.

The treatment must, in a word, comprehend remedies and measures which are indicated by the etiological factors present in every case which comes up for treatment. When the amenorrhea is caused by having contracted cold, the patient should have a warm sitz bath, and hot applications should be applied to the abdomen and thighs. Often a hot vaginal injection will serve a most useful purpose, and a laxative, preferably a saline, will greatly aid in bringing on the flow.

In amenorrhea, delayed menstruation and dysmenorrhea, Ergoapiol (Smith) has acted in my hands in a most satisfactory manner. In scanty menstruation, I found it particularly valuable, and I shall enter in detail about one of a series of cases of this character, later on in this article, where this agent brought on a full menstruation and the general health of the patient began to improve at once. When mental perturbation is a factor in these cases it is manifestly the duty of the physician to have the environments of the patient made as quiet as possible, and antispasmodic or nerve sedatives should be added to the treatment.

When amenorrhea is associated with syphilis, the uric acid diathesis or morbid condition must receive correct treatment. My experience with Ergoapiol (Smith) is such that I regard it as an indispensable remedy in all expressions of amenorrhea along with proper remedies for any diseased condition associated in the causation of the affection. Of course those cases where the amenorrhea is due to atresia of the cervical canal, and to any other condition which is remediable only by surgical means, drugs will prove of no avail. The same can be said of instances in the amenorrhea due to a rudimentary state of the female organs of reproduction.

A lady some time ago brought her daughter to my office for treatment of amenorrhea. The girl was eighteen years old and was visibly anemic. She had an indifferent appetite and was more or less dispirited. She had enough menstrual flow each month to stain the napkin, but this was all that could be said. I had this patient to take Ergoapiol (Smith), one capsule after each meal, and on going to bed regularly for a month. At the next menstrual period the discharge was without pain and free, and the quantity and color was as natural as she had ever known her menstruation to be. She took Ergoapiol (Smith) in the same way another month, and then ceased to have any further trouble. Her color is good and her appetite is likewise excellent; she is full of spirit, and, in a word, well.

A lady aged thirty-three had scanty menstruation which had covered the period of a year. At no time in the year had her menstrual period been longer than eighteen hours, but generally twelve hours told the tale. Her menses were not only scanty, but the color of the menstrual blood was pale, and this was attended with a disagreeable odor. This woman had no associated disease that most searching examination could bring out. Still she had steadily increased in flesh for the last two years, and to this I attributed the amenorrhea.

I had this patient to take systematic exercise and a dietary that was rational, and to take Ergoapiol (Smith) with regularity, a capsule four times a day. After two months this woman ceased to take the remedy, her menstruation having become normal.

A girl twenty years old was sent to me by the matron of a boarding school. She enjoyed good health prior to entering school, but for the past three months she had not menstruated, and was suffering constantly with vertigo and had attacks of hysteria. I attributed the amenorrhea to change of condition of life—that of an open life on a farm to that of a shut-in, inactive life. Ergoapiol (Smith) was given after each meal two weeks prior to the day of her usual menstruation. This brought her menses on fully. She has since had no further trouble in this way.

Mrs. A. P. L., aged thirty-five. This lady suffered with frequent attacks of headache, had backaches nearly all the time, and suffered greatly with vertigo. She was the mother of three children, the youngest being six years old. For the past four years she had constantly had scanty menstruation and the blood was very pale. She rarely had the menstrual flow to continue longer than fifteen hours. I was satisfied that the vertigo and all her distress was due to insufficient menstrual flow, and I accordingly put her on Ergoapiol (Smith). She took it through the month, one capsule after each meal, but for a week before the expected period she took two capsules instead of one. She was greatly pleased this time to have a full and free menstruation. Acting on my advice, she took the capsules three times daily for two months, and this acted in a happy manner, and she has now passed an entire year and has not failed to menstruate freely.

My diagnosis was fully confirmed by this woman's health being good in every way since the establishment of menses on a basis of health.—E. C. WILLEY, M.D., Louisville, Ky., in *The Southern Practitioner*, July, 1902.

